# TWINVAX: conceptual model of a digital twin for immunisation services in primary health care

**DOI:** 10.3389/fpubh.2025.1568123

**Published:** 2025-05-14

**Authors:** L. De Oliveira El-Warrak, C. Miceli de Farias

**Affiliations:** PESC/COPPE - Graduate Programme in Systems and Computer Engineering, COPPE – Graduate School of Engineering, Federal University of Rio de Janeiro (UFRJ), Rio de Janeiro, Brazil

**Keywords:** immunisation, digital twin, vaccines, TwinVax, public health, IoT

## Abstract

**Introduction:**

This paper presents a proposal for the modelling and reference architecture of a digital twin for immunisation services in primary health care centres. The system leverages Industry 4.0 concepts and technologies, such as the Internet of Things (IoT), machine learning, and cloud computing, to improve vaccination management and monitoring.

**Methods:**

The modelling was conducted using the Unified Modelling Language (UML) to define workflows and processes such as temperature monitoring of storage equipment and tracking of vaccination status. The proposed reference architecture follows the ISO 23247 standard and is structured into four domains: observable elements/entities, data collection and device control, digital twin platform, and user domain.

**Results:**

The system enables the storage, monitoring, and visualisation of data related to the immunisation room, specifically concerning the temperature control of ice-lined refrigerators (ILRs) and thermal boxes. An analytic module has been developed to monitor vaccination coverage, correlating individual vaccination statuses with the official vaccination calendar.

**Discussion:**

The proposed digital twin improves vaccine temperature management, reduces vaccine dose wastage, monitors the population’s vaccination status, and supports the planning of more effective immunisation actions. The article also discusses the feasibility, potential benefits, and future impacts of deploying this technology within immunisation services.

## Highlights

- DT appears as one of most discussed technology applications within the Digital Health trend.
- Digital twins hold the potential to transform healthcare, especially in the management and operational efficiency of healthcare services.
- The use of digital twins in primary health care is still poorly explored but has great potential for improving the population’s health indicators, especially across developing countries.
- The use of digital twins in immunisation could support the recovery of vaccination coverage.

## 1 Introduction

Global health has certainly faced its greatest challenge in recent decades. The COVID-19 pandemic pushed health systems to their limits, requiring a rapid expansion of response capabilities. However, in many countries, it became evident that health services failed to meet the rising demands due to an exponential increase in patient volume, inadequacy, unavailability, and insufficiency of all types of resources, technical unpreparedness of healthcare teams, and an overload of, sometimes, inaccurate information, which hindered effective decision-making by managers. Although immunisation is one of the most effective public health interventions, the pandemic placed significant pressure on health systems in 2020 and 2021, resulting in substantial setbacks, particularly with the interruption of routine vaccinations (1, 2).

For over two centuries, the populace has been vaccinated against deadly diseases, ever since the world’s first vaccine was devised against smallpox. History has taught us that a full and effective global response to vaccine-preventable diseases requires time, financial support, and collaboration – and necessitates continued vigilance. Vaccination stands as a remarkable achievement in the realms of medicine and public health, facilitating the eradication of once-devastating maladies. However, this process demands patience and perseverance, involving the coordinated efforts of governments, research institutions, and civil society. Only through this collective endeavour, sustained over decades, can the requisite herd immunity be achieved and maintained for effective protection against vaccine-preventable diseases.

New technologies can play a crucial role in addressing these challenges, providing a solid foundation for quicker and more effective responses during health crises, as well as improving the overall health of populations in normal circumstances. Integrating technological solutions into healthcare is now seen to enable a more efficient and precise approach, improve resource allocation, and strengthen the capacity of health systems to safeguard and enhance public health proactively (3).

Throughout the annals of history, communicable diseases have deeply afflicted human society, resulting in the deaths of countless millions for want of advanced treatments. From the eighteenth century to the present day, many innovations and discoveries have been made in the field of vaccination, enabling the prevention and even eradication of sundry maladies, thereby contributing substantially to the enhancement of the quality of life. Immunisation programs are esteemed as one of the greatest public health successes, warding off the scourge of such diseases as smallpox, polio, tuberculosis, hepatitis B, measles, and rubella, among others, and consequently diminishing mortality rates. Alongside the provision of clean water and improved access to healthcare, vaccinations have played a pivotal role in bolstering life expectancy across many nations (4).

Getting vaccinated is the most effective way to build immunity against vaccine-preventable diseases, rather than relying on immunity gained from contracting the disease itself. Vaccination not only protects individuals from experiencing potentially severe symptoms but also helps prevent transmission by stopping infection when a protected individual is exposed to the pathogen. When high vaccination coverage is achieved within a population, herd immunity can develop, effectively halting the circulation of the pathogen. Moreover, vaccination programs offer broader societal benefits by alleviating the social, psychological, and financial burdens that diseases impose, thereby reducing the strain on healthcare and social care systems and contributing to the overall well-being of communities (5).

The healthcare sector is one of the largest fields of application for new technologies, continually driving innovation (6). Vaccines, however, are highly sensitive products that lose their immunogenicity if not handled correctly. Improper storage and preservation are frequent causes of vaccine waste, as vaccines require strict temperature conditions to remain effective. In this context, technologies such as the Internet of Things (IoT) and Digital Twins (DT) may play a strategic role, even in settings with adverse operational conditions, by contributing to the preservation of essential supplies, despite the constraints imposed by resource scarcity.

In addition to the challenges related to vaccine storage, the decline in vaccination coverage has become a major barrier to improving the effectiveness of primary healthcare interventions. Issues such as a lack of targeted vaccination planning for specific populations, insufficient strategies for tracking individuals who missed doses, limited access to vaccination facilities, supply shortages, and missed opportunities for vaccine administration represent some of the key obstacles to success (3, 7, 8). To enable the implementation of the Digital Twins concept in immunisation, several critical questions need to be addressed. These include determining the appropriate system architecture or design, identifying viable communication protocols for real-time data collection, and selecting complementary technologies for building an effective DT. Solving these challenges is essential for advancing this innovative approach to immunisation.

This work aims to address two fundamental issues: (i) vaccine loss due to inadequate temperature control at storage locations, and (ii) low vaccination coverage in certain target populations. To tackle these challenges, we propose a conceptual framework for a Digital Twin designed for the immunisation service of a primary healthcare centre. Our objective is to illustrate its role in managing the service through the virtualisation of vaccine storage equipment, vaccines, and patients, providing different perspectives on vaccine supply. However, this requires customisation and modelling of various aspects while integrating relevant data into the system.

On the issue of vaccination coverage, the DT builds models to gain deeper insights into the population’s vaccination status. Its ability to explore historical and contextual data becomes an important tool for increasing vaccination coverage. Our main contributions are: (i) A new DT architecture for managing the immunisation service in terms of temperature control of vaccine storage equipment, with capabilities to aggregate and analyse data, and (ii) A module for predicting individualised vaccination needs within a population.

The article is organized as follows: Section 1 consists of the introduction, Section 2 presents related works on Digital Twin applications in healthcare found in recent literature, Section 3 addresses the methodology, explaining the DT modelling, and Section 4 introduces the architecture concept. Section 5 presents the discussions, and finally, Section 6 summarizes the conclusions obtained and outlines future steps.

## 2 Related studies

Vaccination plays a crucial role in advancing the health-related United Nations Millennium Development Goals. The Global Immunisation Vision and Strategy (GIVS), developed by WHO and UNICEF, provides a framework for strengthening national immunisation programs (9). Its objective is to maximize protection against a wide range of diseases by expanding immunisation to all eligible individuals and ensuring that it remains a top priority on health agendas. The strategy aims to achieve or maintain very high levels of immunisation coverage across all age groups, introduce new vaccines, and integrate immunisation with other health interventions. This approach was designed in response to the growing demand for vaccines, rapid advancements in vaccine and technology development, ongoing progress in the health sector, increased vulnerability to pandemics and health emergencies, and expanding opportunities for partnerships (10).

Respecting cultural, economic, and social characteristics, some problems that have a negative impact on achieving optimal immunisation coverage have been more frequent, such as those related to logistics, including delays in receiving vaccines and lack of raw materials in the manufacture of vaccines, storage inadequate and insufficient; to the operation, such as loss of doses due to expiration, non-complete use of doses from open vials, lack of demand for vaccine, work overload of the nursing team, high turnover of professionals in the vaccine room, difficulty of healthcare team in monitoring vaccination control, registration done manually for later entry into the system, difficulty in monitoring the achievement of vaccination targets locations; and structural problems such as lack of personnel to administer the doses, inadequate structure to serve the enrolled population, lack of connectivity or network instability, among others (11–14). Unfortunately, vaccines have, in some way, achieved “victim status” because of their success. Eradication or control of the diseases results in new generations, who have not seen these diseases, underestimating their importance. As a result, some parents start questioning why it is necessary to vaccinate their children- and this questioning can be strong enough that it increases risks for re-introducing or re-emerging previously controlled diseases (or those which were on the verge of being eliminated) (15). This worrisome situation is additionally reinforced by the other problems highlighted – such as vaccine shortages at vaccination centres – with another ally in the form of an influential anti-vaccination movement driven by myths (16).

Certainly, one of the greatest challenges faced by global health is low immunisation coverage, which has contributed to the recurrence of diseases that were previously eliminated. To address this issue, health systems have been challenged to reorganize their work processes, developing innovative strategies combined with the reinforcement of already proved actions, to achieve positive results in expanding the coverage. Immunisation programs require supportive policies and legislation and should set targets for public reporting or benchmarking (17). This involves delivering immunisation programs as part of a broader package of essential health services to maximize reach, minimize missed opportunities for vaccination (MOVs), and make efficient use of resources (18). Immunisation programs in each country may vary in several aspects, such as, age of the individuals to be vaccinated, target population groups, number of doses and the administration schedule, and whether vaccines are administered individually or in combination with others. In many countries, childhood vaccination against certain diseases is mandatory. The differences between them are influenced by factors such as the burden of disease, the structures and resources of healthcare systems, political and cultural factors, as well as the resilience of each program. However, some key points to increase immunisation coverage include a strong national policy with a dedicated budget to boost immunisation efforts and services, training programs for healthcare professionals, and extensive communication with the public about the benefits of vaccination for population health. Ongoing challenges involve developing and implementing strategies to reach underserved populations, supporting evidence-based decision-making for the introduction of new vaccines, strengthening immunisation systems to accommodate these new vaccines, enhancing surveillance of vaccine-preventable diseases, improving the quality of vaccination coverage monitoring, utilizing data to boost program performance, and expanding and improving communication with the public to address information gaps and reduce misinformation.

**Figure 1:**
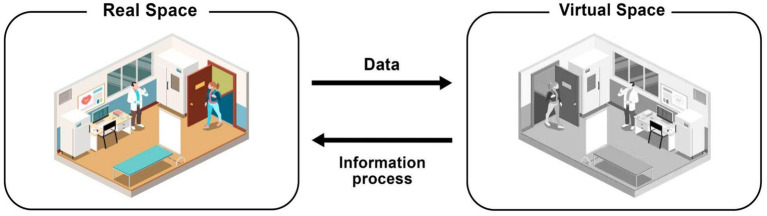
Digital twin representation.

Technological innovations play a central role in the organization of health systems, as they influence how various health services are delivered to the population and the results achieved. These innovations encompass a broad range of technologies used for prevention, diagnosis, treatment, and rehabilitation, such as vaccines, diagnostic kits and reagents, medications, medical and assistive equipment, medical and surgical procedures, and others. Continuous growth and evolution in technologies such as the Internet of Things (IoT), Artificial Intelligence (AI), Machine Learning (ML), 5G, and Big Data have enabled the collection, processing, and storage of substantial amounts of data in real-time. These technologies are essential for the creation and operation of what we now know as the Digital Twin. The concept emerged within 4.0 Industry, aiming not only to simulate real components and systems in a virtual environment but also to integrate these environments through communication protocols, promoting convergence and cooperation between them. A Digital Twin refers to a digital replica of physical assets, processes, people, places, systems, and devices, both potential and real, that can be used for various purposes (19). These replicas function as links between the physical and digital worlds. They gather data over time on a system’s structure, operation, and the environment in which it functions. This data can then be used to build additional intelligence through analytics, physics, and machine learning. As a result, it becomes possible to query a specific system’s digital counterpart to gain insights into its past and current performance, receive early warnings and predictions, and enhance productivity. To be effective, a Digital Twin must meet at least three prerequisites: first, a physical object or asset to be replicated; second, a virtual version of this object must be created; and third, a continuous connection between the physical object and its virtual counterpart must be maintained through data collection. This ensures that the virtual representation remains up to date. The Figure 1 illustrates a digital twin representation:

The concept of digital twins originated in Industry 4.0, where its applications have been extensively explored and refined. Tao et al. (20) highlight how digital twins are applied across key phases of the product lifecycle—design, manufacturing, and service. Product Lifecycle Management (PLM) has emerged as a particularly promising area for digital twin adoption, drawing significant interest from both industry and academia. Despite these advancements, the field still faces technical challenges, as outlined by Tao and Zhang (21), who proposed a digital twin architecture for the manufacturing floor. Their study points to issues such as real-time connectivity, model consistency, and large-scale data handling. Likewise, Uhlemann et al. (22) developed an architecture suited for small and medium-sized enterprises, emphasizing difficulties like data standardization and real-time synchronization, while Zheng et al. (23) applied a digital twin framework to a welding station, tackling data collection, virtual modelling, and real-time processing.

Building on the successful adoption of digital twins in industry, there is growing interest in their application within healthcare, where numerous studies are underway. In healthcare, digital twins hold the potential to transform how patient data is gathered, analysed, and used for personalized care. Individuals generate real-time data through wearable devices and sensors, tracking vital signs, physical activity, and environmental factors. Combined with electronic health records, these data sources offer valuable insights, enabling both caregivers and patients to monitor vaccinations, medications, and health trends. Some researchers refer to individuals as “walking sensor platforms” (24) or “data beings” (25), illustrating the vast data potential that digital twins can harness in healthcare.

The integration of digital technologies into the immunisation process offers a promising pathway to overcoming some of the operational challenges faced by vaccination programs. Digital Twins, for example, can provide valuable insights into the efficient management of vaccine stocks by monitoring, in real time, the temperature of cold storage units and the distribution of vaccines across healthcare facilities. Additionally, these systems can be integrated with population behaviour data, enabling the prediction and mitigation of risks associated with low vaccination adherence in specific communities. This intelligent use of data allows for more efficient resource management, directing intensified vaccination efforts toward more vulnerable areas or groups, thus increasing coverage, and ensuring that public health goals are met.

Studies have further highlighted the use of digital twins in diagnosing conditions, simulating therapies, and predicting treatment outcomes on individual and population levels. In one example, Liu et al. (26) developed a cloud-based solution using digital twins to enhance older adult care services, highlighting the transformative potential of digital twin technology within healthcare.

Chaudhari et al. (27) discussed how Digital Twin technology enhances digital health monitoring systems within the context of Industry 4.0. It describes Digital Twins as real-time virtual replicas of physical objects, enabling continuous health tracking and analysis. By leveraging the Internet of Things (IoT) and artificial intelligence, Digital Twins provide valuable insights and predictive analytics. This approach aims to improve healthcare efficiency and patient outcomes. The authors emphasize that integrating Digital Twin technology can significantly advance adaptive healthcare systems.

Katsoulakis et al. (28) examined the applications of digital twins in healthcare, highlighting their role in enhancing personalized treatment strategies and improving patient outcomes. The authors emphasize the integration of computational models and artificial intelligence, which enable real-time monitoring and data analysis in clinical settings. Their analysis categorises various applications of digital twins, showcasing their potential to transform healthcare delivery.

Aubert et al. (29) explored the application of digital twins to improve trauma surgery and postoperative management, specifically in tibial plateau fractures. The authors develop a digital twin model that simulates patient anatomy and surgical interventions, enabling better preparation and planning.

Björnsson et al. (30) examined the use of digital twins as an innovative tool for personalized medicine. The central idea is to create digital replicas of patients by using large volumes of biological, clinical, and behavioural data, thus allowing for simulation and prediction of individual responses to treatments and medical interventions.

Stahlberg et al. (31) investigated the potential of digital twins in cancer prediction. The authors discuss various approaches to creating models that can simulate disease progression and treatment response, aiming to improve therapeutic planning and clinical outcomes.

Popa et al. (32) analysed the social and ethical impacts of implementing digital twins in healthcare, raising concerns about data privacy, equitable access to this technology, and potential algorithmic biases.

Sahal et al. (33) discussed the use of emerging technologies, such as digital twins, blockchain, the Internet of Things (IoT), and Artificial Intelligence (AI), to address pandemic crises. They propose a blockchain-based collaborative framework for decentralised epidemic alerts, aiming to combat COVID-19 and future pandemics, and emphasize the importance of secure, real-time data exchange among multiple actors to support COVID-19 response efforts.

Elkefi and Asan (34) highlighted the main applications of digital twins in healthcare, such as clinical process optimization, patient monitoring, and resource management. The study also addresses the challenges and limitations in implementing this technology, including issues of privacy, data integration, and costs.

Madubuike and Anumba (35) investigated the use of digital twins to enhance healthcare facility management. The authors focus primarily on civil engineering aspects of healthcare facilities, aiming to improve preventive maintenance and other applications to optimise management processes. They also propose a digital twin system architecture for these applications.

According to a literature review conducted by de Oliveira El-Warrak and Miceli de Farias (36), the main applications of digital twins in healthcare can be divided into two categories: clinical and operational applications. Clinical applications include personalized care, through the creation of patient-specific models, and the reproduction of biological structures, such as tissues and organs, to simulate treatments and interventions. Operational applications involve the optimization of processes, such as patient flow and resource management, as well as the reproduction of physical structures, like hospital layouts, and the development of devices and medications. Despite challenges related to data integration, privacy, and interoperability, the use of digital twins promises significant advancements in treatment personalization, remote monitoring, prevention, and decision-making, with a positive impact on healthcare quality and efficiency.

de Oliveira Ribeiro et al. (37) proposed the development of a digital twin architecture aimed at digitising and modernising Brazil’s National Vaccination Plan in response to the COVID-19 pandemic. This technology aims to improve resource management, allowing real-time simulations and analyses. This enables quick and informed adjustments, increasing transparency and efficiency in the allocation of public resources in healthcare, specifically in immunisation. In another study on the use of digital twins in healthcare facilities, de Oliveira Ribeiro (38) proposed a maturity model to evaluate the readiness of public healthcare units in Brazil for implementing digital twins (DTs). By analysing critical success factors found in the research, the study highlights the importance of elements such as Modelling and Simulation, Information Security, Logistics, Structure, and Organizational Management for successful DT implementation.

From the reviewed literature, a handful of key points can be inferred. Firstly, most studies on digital twins (DTs) are theoretical, focusing on opportunities and challenges of use or mentioning DTs as a promising technology for healthcare, revealing a need for more practical applications of this technology for subsequent evaluation.

Secondly, a few studies address Digital Shadow or Digital Model rather than data exchange between the physical entity and the DT, limiting the exploration of certain DT potentialities, such as acting on the real system following intelligent analyses in the virtual model, as highlighted by Cimino et al. (39).

Thirdly, both Cimino et al. (39) and Tao et al. (40) observed that even in DT-focused studies, the virtualized element is typically only an asset, without a comprehensive view of the system in which it is embedded.

Fourthly, most studies on DTs in healthcare operations are simulated rather than connected to a real system. This simplifies the complexity of integration and interoperability aspects and excludes human operator actions, limiting the studies to equipment simulations (29, 30, 34, 39, 40).

Finally, regarding DT architectures, while some studies list functionalities for a complete DT system, there are a lack of concrete proposals for architectures or methodologies to assist in the implementation of DTs in the ecosystem addressed in this study, specifically in Primary Health Care.

## 3 Modelling the digital twin in immunisation

### 3.1 Characterisation of the physical system–vaccination room

Primary Health Care (PHC) is the most strategic level of care for the prevention of diseases and health conditions, standing out for being, among its essential attributes, the first point of contact for users of the health system. Thus, from the perspective of the control, eradication, and elimination of vaccine-preventable diseases, which includes immunisation activities, it is essential that PHC has adequate and responsive structures. The local level of the health system plays a crucial role in shaping, acting, and taking responsibility for PHC activities, including the planning and organisation of vaccination within communities (3, 7, 17, 18). This level is responsible for providing suitable locations for the storage and administration of vaccines, as well as for making available trained nursing professionals for the proper management, maintenance, and handling of immunobiologicals. It also plays a key role in supporting the monitoring and evaluation of immunisation activities within vaccination rooms. Several vaccination strategies were implemented in Primary Health Care to reach susceptible populations, including the showed-on Table 1.

**Table 1:** Vaccination strategies–adapted from World Health Organization (8).

Strategies	Description
Routine vaccination	A systematic approach to vaccination aimed at controlling diseases preventable by vaccines through widespread coverage.
Routine vaccination intensification	Targeting individuals who have not been vaccinated or have not completed their vaccination doses.
Rapid monitoring of immunisation coverage (RMC)	A method to assess vaccination coverage and identify unvaccinated individuals within susceptible populations by checking vaccination records, such as child vaccination cards, during homecare visits.
Catch-up vaccination	Action of vaccinating an individual who, for whatever reason, is missing or has not received doses of vaccines for which they are eligible, per the national immunisation schedule.
Community-wide vaccination	Conducted when there are one or more suspected cases of a disease with a high potential of spread
Ring vaccination	Used to control the spread of infectious diseases. It involves identifying cases of the disease, then vaccinating and monitoring all the contacts and contacts of contacts around each case to form a ‘ring’ of immunity and prevent further spread

In primary care centres, the vaccination room is the designated area where vaccination procedures are carried out, whether through routine actions, campaigns, or other strategies, and is classified as a semi-critical area. To ensure maximum safety and minimize contamination risks for both vaccinated individuals and vaccination staff, it is essential to adhere to specific requirements and conditions regarding the environment and facilities. There is no universally recognised standard measurement for vaccination rooms, as dimensions may vary based on local regulations, the number of vaccines administered, and available space. However, many guidelines suggest that a common vaccination room should be between 10 to 15 square meters, which is adequate to include a waiting area, a vaccination area, and space for equipment. The waiting area should accommodate patients and their companions, typically ranging from 5 to 10 square meters, while the vaccination area requires space for furniture and equipment, averaging around 10 square meters (17, 41, 42). Ideally, the vaccination room should be exclusively dedicated to the administration of immunobiologics and, if possible, have independent entry and exit points. The basic equipment for these rooms includes a counter for preparing vaccines, an ice-lined refrigerator (vaccine refrigerators) for their storage, a table with drawers, chairs, a paper towel holder, a cabinet for storing necessary materials, and surgical instruments. Key supplies include clinical thermometers with extension cables, reusable ice packs, disposable syringes, disposable needles, vaccine carrier boxes (or coolers) - particularly in case of power outages or during outreach vaccination activities - and for transporting vaccines, among others. In some vaccine rooms, thermal boxes are also used to temporarily store vaccines during the unit’s operating hours. This practice helps to prevent the frequent opening of the cold storage units, which could lead to temperature fluctuations and loss of the optimal storage conditions for the vaccines.

According to the CDC (42), vaccines are biologically sensitive substances that gradually lose their potency, or effectiveness, when exposed to temperatures outside the recommended storage range. Once potency is diminished, it cannot be restored, even if the vaccine is returned to proper storage conditions. This loss is irreversible and permanent. Therefore, it is essential to store vaccines under the correct temperature conditions to preserve their full potency until the point of administration. Vaccines lose potency when exposed to heat (temperatures above +8°C), cold (temperatures below +2°C), or light. Reconstituted vaccines such as measles/MR, and Japanese encephalitis (JE) are particularly sensitive to heat and light. Since these live vaccines lack preservatives, they carry a risk of contamination with *Staphylococcus aureus*, which can lead to toxic shock syndrome; therefore, they must be used within 8 h of reconstitution. These light-sensitive vaccines are supplied in amber-coloured vials to reduce light exposure (41, 42).

Ideally, all vaccine storage units should be equipped with a Temperature Monitoring Device (TMD). Maintaining an accurate temperature history that reflects the actual vaccine temperatures is critical for preserving the integrity of the vaccines. Investing in a reliable TMD is a prudent measure, as it is less expensive than replacing vaccines that have lost potency due to storage at out-of-range temperatures. Regarding internal organization, it is essential to store vaccines in properly refrigerated facilities and equipment to prevent the inactivation of the immunobiologics. Vaccines should be arranged in a manner that allows for adequate air circulation among the products or stored boxes (42).

To ensure proper vaccine storage and minimize the risk of administration errors, the following practices listed in Table 2, should be adhered to:

**Table 2:** Storage and handling practices for vaccines and diluent management–adapted from CDC (42).

Practice	Description
Storage in original packaging	Each vaccine or diluent must be stored in its original packaging and in separate containers.
Distance from walls, ceiling, floor, and door	Vaccines and diluents must be positioned 5 to 7 cm (2 to 3 inches) away from the walls, ceiling, floor, and door.
Organisation on shelves	They must be arranged in rows with space between them to promote air circulation.
Clear labelling	Shelves and containers must be clearly labelled to identify the specific location of each vaccine and diluent.
Separation of similar vaccines and diluents	Vaccines and diluents with similar packaging, names, or formulations (e.g., paediatric, and adult versions) must be stored on separate shelves.
Storage of diluents	Whenever possible, diluents should be stored with their corresponding refrigerated vaccines and must never be stored in a freezer.
Limitation of non-vaccine items	Non-vaccine items must be limited within the storage unit. If other medications or biological products are necessary, they must be clearly marked and stored in separate compartments.
Prioritisation by expiration date	Vaccines and diluents must be prioritised by expiration date, with those expiring sooner placed at the front to ensure proper stock rotation.

An Ice Lined Refrigerator (ILR) is designed for storing vaccines safely, maintaining an internal temperature of +2°C to +8°C. It requires only 8 h of power within a 24-h period, making it a reliable choice for vaccine storage under the Universal Immunisation Programme (UIP) at Primary Health Centres (PHC) 43. Ice packs, prepared in a deep freezer, are used to extend cooling in vaccine carriers and thermal boxes. It is noteworthy that integrating real-time temperature monitoring systems can significantly enhance the reliability of storage conditions by enabling the prompt detection of temperature deviations and facilitating timely interventions to maintain vaccine integrity (17, 41, 42). Additionally, to prevent temperature loss from the frequent opening of the ILR, thermal boxes are used in vaccination rooms to temporarily store vaccines during their use. This practice helps ensure that the ILR remains closed, maintaining its optimal temperature range while facilitating the efficient distribution and administration of vaccines.

Regarding the vaccination team, activities are carried out by professionals who are properly trained and qualified in the handling, conservation, preparation, and administration of immunobiologicals. These professionals are also responsible for patient triage and for recording data in the Vaccination Information System. Typically, the vaccination room is staffed by a nurse, a nursing technician, and, in some primary healthcare units, administrative personnel. However, it is important to note that this staffing configuration is dependent on the specific regulations of each country and cannot be generalised across different healthcare systems. Among the key activities needed to monitor the safety of immunobiologicals in relation to their handling and storage conditions are the following:

1. *Verification of Equipment Temperature*: Prior to opening the vaccine storage equipment, it is essential to check its temperature. Immunobiologicals should be maintained between +2°C and +8°C.
2. *Preparation of Reusable Ice Packs*: Ice packs come out of the deep freezer (DF) at a temperature of about −20°C. It is essential to remove the reusable ice packs from the freezer and allow them to sit on the counter for several minutes until they reach a positive temperature of approximately +1°C before organizing them in the thermal box. This process, known as “conditioning,” is necessary to ensure that the ice packs effectively keep the desired temperature for vaccines during transport.
3. *Organization of Daily Use Thermal Box*: The thermal box should be selected for daily use, with acclimated ice packs arranged around the inner walls of the box. Positioning the probe of the thermometer inside can ease effective temperature monitoring. Once the temperature is in the proper range, vaccine vials can be placed in a container, ensuring they do not come into direct contact with the ice packs. This arrangement supports optimal preservation of the vaccines during transportation.
4. *Control of Vaccine Quantity*: It is recommended that the number of vaccines in the thermal box be approximately sufficient for the workday, which can help minimize frequent openings of the ILR.
5. *Temperature Recording*: It is advisable to conduct a sequential verification of temperatures, starting with the current temperature, followed by the maximum and minimum temperatures.
6. *Daily Monitoring and Evaluation*: It is recommended to monitor and evaluate the temperature status daily, categorising it as alert, ideal, or inadequate. Any deviations from established limits (below +2°C or above +8°C) should be communicated immediately to the responsible for the vaccination room.
7. *Proper Disposal of Materials*: Designated collection boxes should be utilized for each type of material to be discarded, ensuring the separation of live vaccines, inactivated vaccines, and syringes/needles. Keeping the collection boxes away from the trash and sink can help prevent contact with moisture.

At the end of the day, it is advisable to disassemble the thermal boxes, clean, and dry them, preferably leaving them open. Organizing the immunobiological vials within the ILR for subsequent use is recommended, ensuring that the validity of each product is respected following its opening. Ice packs should be cleaned and stored in the DF.

Regarding the registration, monitoring, and control of patients attended and doses administered, it is essential to request a unique identifier for the user. This identifier enables the digital consultation of the vaccination history within a dedicated database, with the user’s personal data being verified. In cases where the digital record cannot be found, any physical documentation of the vaccination history, such as a vaccination booklet or other proof, must be requested for evaluation. If the user is attending for the first time, the nominal vaccination registration documents must be opened in the computerised system, using the unique identifier.

A brief clinical anamnesis should be conducted to assess any adverse events related to previous vaccine doses, current medication use, history of allergies to any substances, and the user’s current health status. When registering new doses, it is crucial to enter the primary data, including the identifier, vaccination details, and any necessary observations, prioritising real-time data entry. If real-time entry is not feasible, the registration must be completed within 48 h following the vaccination procedure.

Vaccination is a crucial public health intervention, essential for substantially reducing morbidity and mortality from preventable diseases worldwide. Therefore, vaccination rooms must be appropriately structured and equipped with adequate physical, material, and human resources to ensure safe and high-quality care. Furthermore, continuous education for professionals in these settings is imperative. As new vaccines are introduced and scientific knowledge advances, it is critical to train those responsible for vaccine storage and administration. While investing in education is essential, it is equally important to implement procedures that guarantee the practical application of acquired knowledge, aligning daily practices with established standards. This approach enhances the quality of vaccination services, ultimately benefiting the population by ensuring high-quality care (17, 41–43).

### 3.2 Creating the digital twin for immunisation service

#### 3.2.1 Conceptualisation

A model is understood to be a representation of a real system, utilized for the purpose of conducting simulation studies. All elements pertinent to the capture of the desired information that constitute the real system must be included within the model. Consequently, the system under investigation should possess an accurate representation, thereby ensuring the quality of the information derived from the simulation (44, 45). The utilization of discrete event simulation (DES) in this study is warranted by the characteristics of the data intended for use in the modelling of the digital twin. In this type of simulation, data assumes discrete values with clearly defined transitions. Furthermore, in DES, all data, entities, and activities are identifiable once the model is finalized, allowing for a chronological understanding of events. The core of a DT comprises virtual models. Thus, the most critical aspect of constructing a DT is the development of high-precision virtual models that accurately represent the physical properties, behaviours, and rules of the real object. In general, the creation of a digital twin encompasses two primary areas of concern: (i)- Developing the digital twin processes and information requirements throughout the product life cycle - from the design of the asset to its field use and maintenance in the real world, and, (ii)- Creating the enabling technology to integrate the physical asset with its digital twin, allowing for the real-time flow of sensor data, as well as operational and transactional information from the organization’s core systems, as outlined in a conceptual architecture.

This study proposes a digital twin characterized by four key features: Modelling and Simulation, Real-time Data Integration, Analysis and Optimization, and Insights and Action. Modelling encompasses a comprehensive representation of the physical object or system, integrating various attributes such as mechanical, electrical, and physical properties. Simulation involves subjecting the digital twin to diverse conditions to forecast its behaviour under similar scenarios in the real world. Real-time data integration utilizes IoT sensors to gather continuous data from the physical counterpart, enhancing the simulation’s precision and facilitating early detection of issues. Furthermore, advanced analytical techniques and machine learning algorithms can be employed to discern patterns, anticipate failures, and propose enhancements.

The use of real-time or near-real-time physical data is essential for updating these virtual models and simulating the physical process and its evolution. The network plays a crucial role in connecting the physical object (PO) to its virtual representation (VO). This connection facilitates real-time data collection and the bidirectional exchange of control commands. Data is transmitted in two directions: data from the PO updates the state of the virtual representation, while information from the virtual representation is sent back to the physical system. As a result, the insights and decisions generated by the virtual representation provide feedback to the physical system. Typically, communication between the PO and VO involves three main stages (46): (i) collecting the necessary information, which includes the direct measurement of physical reality; (ii) interpreting the data according to the required level of abstraction, which may involve processing, curating, and converting the data; and (iii) using this data to update the system states. This may require integrating information from multiple sources. The interface that establishes the connection between the real process and the Digital Twin, represented by the simulation model, can be designed, and implemented in various ways, depending on the characteristics of the simulation software and the connectivity capabilities of the real systems.

A two-phase approach can be employed for predictive modelling in health services management. The first phase emphasizes offline development, utilizing Machine Learning (ML) and Deep Learning (DL) techniques, such as classifiers, to construct and train the model using historical data from the Digital Twin (DT). In this controlled setting, the model enhances its accuracy through existing data before deployment in real-time applications.

The second phase entails the online deployment of the trained model at the edge, near the data source, to minimize latency and optimize performance. By leveraging real-time streaming data from the DT, the model can identify potential risks promptly, enabling swift responses and necessary adjustments. This two-phase methodology integrates comprehensive historical data analysis with the agility of real-time data processing, ensuring a robust and reliable predictive model for proactive health services management. Optimization occurs by refining parameters in the digital model and implementing best practices in the physical realm. Insights derived from the digital twin subsequently inform enhancements to health services, creating a positive feedback loop that drives improvements in service delivery (47, 48).

**Figure 2:**
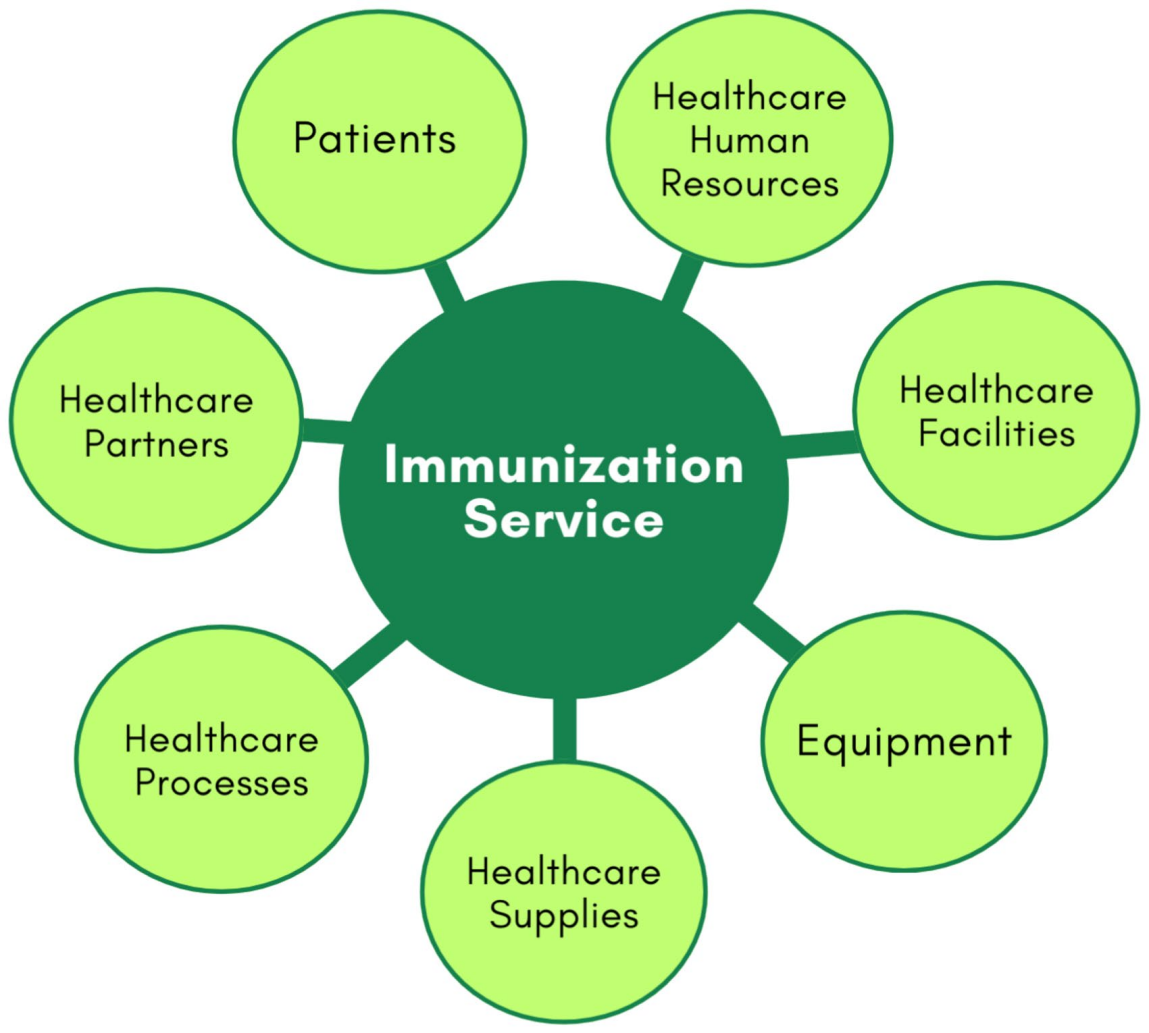
Main components in healthcare process.

For modelling, an immunisation service in Primary Healthcare Centre can be divided into seven main components or entities, as illustrated in Figure 2. These components include patients, health human resources, facilities, equipment, health supplies, processes, and partners (49).

The patients’ component includes various types of patients, categorised by age groups, health histories, and specific needs, such as those with acute and chronic diseases, disabilities, or immunological risks. The healthcare human resources encompass nurses, technicians, and operational staff. Healthcare facilities cover the immunisation room, waiting area, and staff offices. Equipment pertains to all medical devices, IT infrastructure, and furniture. Healthcare supplies are divided into physical and service supplies. Physical supplies include vaccines, medications, drugs, lab materials, cleaning supplies, treatment materials, and other essentials for maintaining healthcare facilities and equipment (41). Service supplies consist of crucial services received from partners, such as maintenance for medical equipment, catering for staff, patients, and visitors, and utilities like energy and water. Processes include procedures for treating patients with immunobiologicals, managing medical emergencies, organising the vaccine room, staff scheduling, recording information in systems, inventory monitoring of vaccines, supply chain management, workflow optimisation, and other operational processes. Partners include suppliers of equipment and consumables, hospitals, specialized healthcare centres, and others.

Digital twins can be created for all these components. They use data from healthcare facilities, equipment, processes, patients with various needs, supplies, and partners, compiling real-time information from sensors, health information systems, such as electronic medical records (EMR), electronic health records (EHR) and other sources to create digital replicas. For example, digital counterparts can be developed for healthcare facilities such as X-ray rooms and other healthcare processes such as treatment and logistics processes.

On the other hand, creating DTs of patients presents one of the most complex challenges in healthcare due to the need to represent diverse patient characteristics such as age, gender, health history, current health status, and healthcare needs. Studies such as those by Okegbile et al. (50) have explored the main design requirements and enabling technologies of digital patient twins, as well as the technical challenges present. The complexity involved stems from multiple levels of abstraction, different types of patients, numerous environmental factors, and continuous and rapid changes in healthcare data. Patient digital twins can be developed with varying levels of detail depending on their purpose. For example, refined models can reflect real-time health and environmental information from individual patients, supporting personalized medical services. For most applications in immunisation services, these detailed individual models are extremely valid. However, even if there is no detailed health analysis, the immunisation service also benefits from an abstract view of aggregated patient data to support high-level decision-making, improving overall efficiency, quality, access, and the cost-effectiveness of vaccination. This model comprises (i) a patient information database populated with clinical data from multiple sources; (ii) a cloud computing platform; (iii) traceability systems using AI; and (iv) blockchain technology. Other components, such as human resources, facilities, and equipment, are less complex and can be generalised based on their specific characteristics. For instance, a digital twin of a nurse would focus on their schedule, work location, and skills, rather than individual traits. Similarly, digital twins for facilities and equipment are relatively static and can be periodically updated as needed.

This study will focus on three components: the equipment used for vaccine storage, the vaccines themselves, and the patients. For the equipment entity, the monitoring of operational conditions will be based on the temperature attribute. In the case of the vaccine entity, monitoring will be conducted based on the type and number of doses, while for patients, it will pertain to their vaccination schedule and history. The DT will function by providing alerts regarding variations in the ideal temperature conditions for storage that may jeopardize the vaccine’s efficacy, as well as alerts for timely vaccination needs for patients.

Furthermore, the digital twin should propose scenario analyses for individuals and/or groups who may delay or miss certain vaccines. Additionally, considering the vaccination needs according to the vaccination schedule of the target public, the DT could also estimate the ideal quantity of vaccines to be available in the immunisation room. In this work, the events of interest will include the temperature measurements collected by sensors and the vaccines administered to patients by dose and type, as recorded in the information systems designed for this purpose.

#### 3.2.2 Key concepts and principles in digital twin modelling

The proposed modelling focuses on the primary entities related to immunisation services, including patients, healthcare supplies (Vaccines), and equipment (such as ILR and thermal boxes or carrier boxes), as well as the relationships among these entities. The primary objective is to ensure the monitoring of the conditions of vaccines stored in equipment, specifically concerning temperature, thereby safeguarding the integrity of the vaccines during storage. Furthermore, the modelling will facilitate predictive analysis of vaccination coverage among patients, optimizing the planning of preventive actions based on a vaccination schedule. Other entities, such as processes, facilities, and health human resources, will not be addressed in this work, as they pertain to operational aspects outside the current scope.

This work aims to construct a DT of an immunisation service, which will enable real-time monitoring of vaccine temperatures within equipment and provide simulations to enhance vaccination coverage. By leveraging information on administered vaccines and the need for vaccination according to the vaccination schedule outlined in the calendar, healthcare teams can adopt a proactive approach to immunisation. This system will facilitate the identification of individuals with upcoming vaccination dates and issue alerts to patients regarding the necessity of receiving their vaccines within the specified timeframe. It should be noted that this first configuration of the DT does not include control of the stock of vaccines, which could be done later.

**Figure 3:**
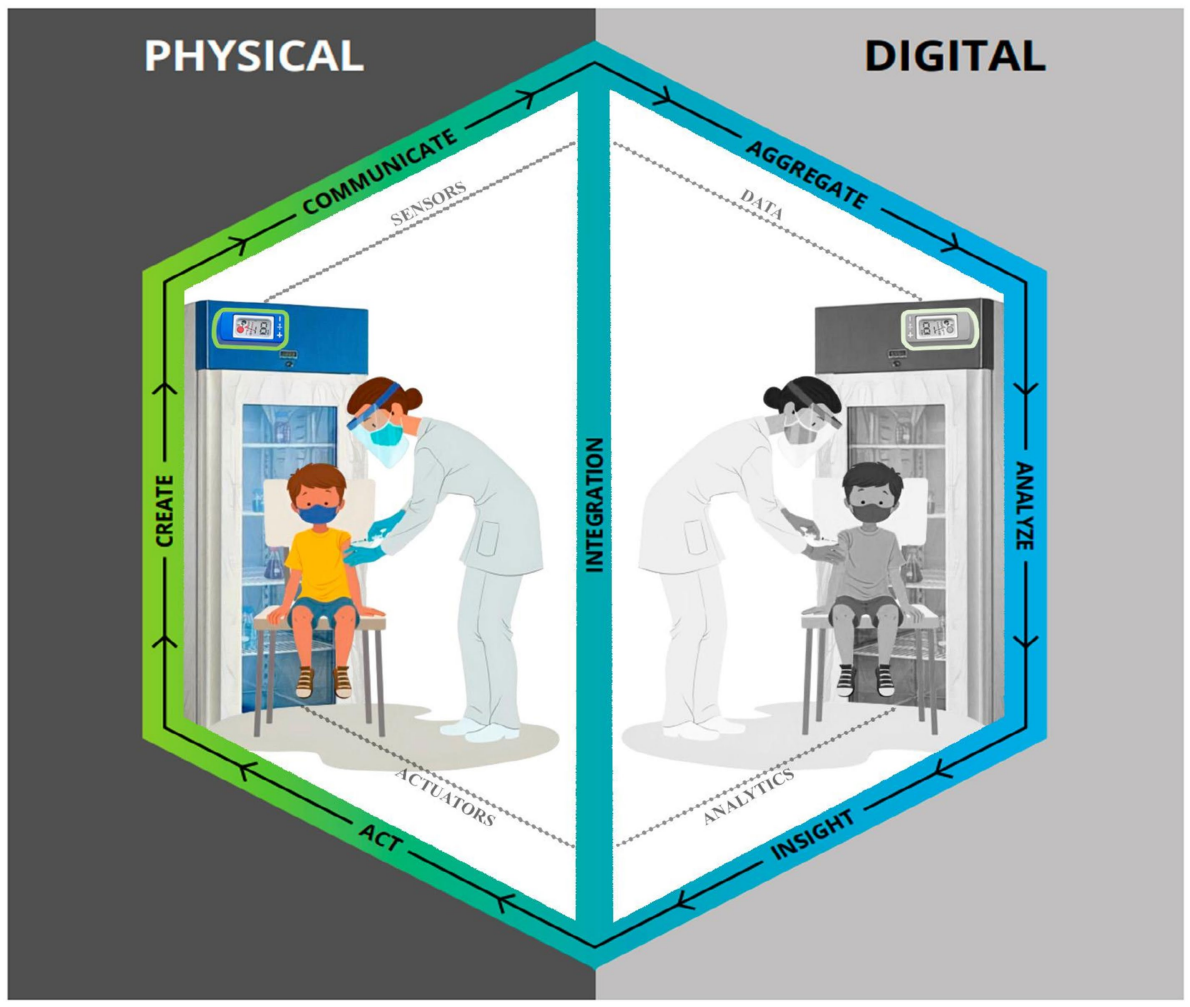
Digital twins modelling steps by Parrott and Warshaw (54).

The main components needed to build a digital twin are sensors/actuators, historical process data, connection between the physical and the digital worlds and data analysis. The data from the sensors are integrated to the historical data, the simulation runs in real time, mirroring the physical state, and the data analysis decides the actions that need to be taken (51, 52). A digital twin also needs a model that describes the real world. For Kaur et al. (53), the basic design of a DT consists of sensor technologies and specificities, IoT (Internet of Things) and machine learning. From a computational perspective, data and information fusion is the key technology for implementing the digital part, thus facilitating the flow of information from raw sensor data to high-level understanding and insights. Therefore, it is possible to consider that digital twins operate based on two main points: communication and data. While the foundational principles are applicable across various digital twin configurations, according to Parrott and Warshaw (54), modelling a digital twin must necessarily involve the following steps illustrated in Figure 3 and described below:

1 Creation: The creation phase involves equipping the physical process with a variety of sensors that capture crucial inputs from both the process itself and its environment. These sensor measurements can generally be categorised into two main types: (i) operational metrics related to the physical performance standards of the productive asset (alongside various works in progress), which include factors such as tensile strength, displacement, torque, and colour consistency; and (ii) environmental or external data that impact the asset’s operations, such as ambient temperature, barometric pressure, and moisture levels. These measurements can be converted into secure digital messages using encoders and subsequently sent to the digital twin. Additionally, the signals from the sensors can be enhanced with process-related information from systems like manufacturing execution systems, enterprise resource planning systems, CAD models, and supply chain systems. This combination provides the digital twin with a comprehensive array of continuously updating data to be utilized for its analysis.
2 Communication: The communication phase facilitates seamless, real-time, bidirectional connectivity between the physical process and the digital platform. Network communication represents one of the fundamental changes that have enabled the digital twin concept; it comprises three primary components:

- Edge Processing: The edge interface acts as a bridge between sensors and process historians, enabling signals and data to be processed close to their source before being transmitted to the main platform. At this stage, proprietary protocols are converted into more accessible data formats, minimizing the need for large-scale network communication. Recent advancements in this area have addressed many previous limitations affecting the effectiveness of digital twins.
- Communication Interfaces: Communication interfaces are crucial for transferring information from sensor functions to integration functions. A wide variety of options is essential due to the ability to position the sensors that generate insights almost anywhere, depending on the specific configuration of the digital twin in question. This can include locations such as factories, residences, mining operations, or parking lots, among many others.
- Security at the Edge: The introduction of new sensor and communication technologies has presented several security challenges that continue to evolve. Common security approaches include firewalls, application keys, encryption, and device certificates. As more assets connect via Internet Protocol (IP), there is an increasing need for innovative solutions to implement digital twins securely.

3 Aggregation: The aggregation phase involves collecting data for storage in a repository where it can be processed and prepared for analysis. This aggregation and processing of data can occur locally or in the cloud. Recent technological advancements have enabled the development of highly scalable architectures that are more agile and cost-effective than ever before.
4 Analysis: In the analysis phase, the collected data undergoes examination and visualization. Data scientists and analysts leverage advanced analytical platforms to create iterative models that provide insights and recommendations, aiding the decision-making process.
5 Insights: During the insights phase, the outcomes of the analysis are presented through dashboards featuring visualizations. These visual representations highlight significant discrepancies between the performance of the digital twin and its physical counterpart across various dimensions, pinpointing areas that may require further investigation and adjustment.
6 Action: The action phase involves relaying actionable insights from previous steps back to the physical asset and the digital process, allowing the digital twin’s full potential to be realized. Insights are processed through decoders and sent to the asset’s process actuators, managing movement or control mechanisms. Alternatively, this information can be updated in back-end systems overseeing supply chain management and ordering behaviour, always under human oversight. This exchange effectively closes the loop between the physical environment and the digital twin.

Reference modelling in digital twins remains an emerging field, with the layered approach being the most adopted methodology. In order to standardise a DT implementation architecture framework for our application in an immunisation service, the ISO 23247 standard will be used. Officialised in 2021, the latter was developed by the ISO TC 184/SC 4 committee with the aim of establishing a framework for implementing Digital Twins in industrial manufacturing applications, in the context of Industry 4.0 (45).

#### 3.2.3 UML modelling

UML (Unified Modeling Language) plays a fundamental role in the conceptual and structural configuration of a digital twin, particularly during the initial phases of system planning, specification, and validation. It provides a standardised approach to representing and communicating the components, logic, and interactions that underpin the connection between the physical system and its digital counterpart. Through class and component diagrams, UML helps specify key system elements—such as sensors, devices, data repositories, machine learning models, and communication interfaces—while sequence and activity diagrams enable the modelling of data flows and system behaviours. Moreover, UML facilitates the abstraction of layered architectures, making it easier to distinguish between the sensing layer, connectivity, modelling, and application domains. In the context of digital twins in healthcare, UML also serves as a foundation for in silico simulation, enabling the verification of the proposed model’s logic and behaviour prior to any real-world implementation. By providing clarity, standardisation, and traceability, UML strengthens the development of digital twins as complex socio-technical systems.

**Figure 4:**
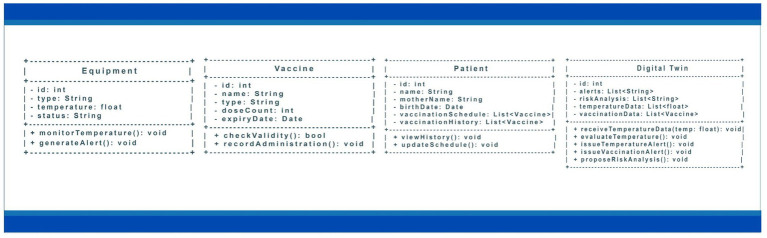
Classes, attributes, and methods in the proposed UML modelling.

The UML modelling of the proposed system is centred on the entities previously defined as the scope of the research: Equipment, Vaccine, Patient and Digital Twin. The interaction between these elements is designed to ensure the proper conservation of vaccines, the organisation of patient information and data-based decision support for the administration of immunobiologicals. Figure 4 illustrates the UML modelling class proposed for the immunisation service of a Primary Healthcare Centre.

The Equipment class represents essential devices for preserving vaccines, such as ILRs and thermal boxes. This class has attributes such as id, which guarantees the unique identification of each equipment, type, which categorises the equipment, and temperature, which records its current thermal condition for preserving vaccines. In addition, the status attribute provides information on the equipment’s operational status, indicating situations such as normal operation or the need for maintenance. The methods include monitorTemperature(), which constantly reads the temperature to maintain ideal storage conditions, and generateAlert(), which is responsible for issuing notifications in the event of deviations from tolerable limits.

The Vaccine class is intended for vaccination control and the specific characteristics of each immunobiologicals. Its attributes include the id, which identifies each vaccine, the name, which specifies the immunobiologicals, and the type, which classifies it as a routine, booster, or campaign vaccine. In addition, the numberDoses, which defines the number of doses required, and the expiry date, which ensures that only vaccines within the expiry date are administered, are recorded. The methods in this class include checkValidity(), used to prevent the administration of expired vaccines, and registerApplication(), which updates the patient’s vaccination history after administration.

The Patient class organises the data relating to the individuals taking part in the immunisation process. The main attributes include id, name, mother’s name, and date of birth, which allow the patient to be accurately identified. In addition, the vaccinationSchedule and vaccinationHistory attributes are used, which store, respectively, the vaccines scheduled for application and those already administered. Methods such as viewHistory() allow access to the vaccination history, while updateSchedule() allows adjustments to the vaccination calendar based on changes in requirements or applications carried out.

The Digital Twin class forms the core of the system, integrating and monitoring information related to the other components. Its attributes include id, which identifies it, alerts, which store messages about critical situations, and risk analysis, which record the results of the assessments carried out. In addition, the dataTemperature and dataVaccination attributes provide a comprehensive view of the storage conditions and vaccination history of patients. Methods such as receiveTemperatureData() and evaluateTemperature() ensure continuous monitoring of thermal conditions, while issueTemperatureAlert() is triggered when critical values are recorded. In turn, issueVaccineAlert() notifies of upcoming vaccination dates, and proposeRiskAnalysis() supports the prioritisation of actions based on epidemiological criteria.

The relationships between the classes reinforce the system’s integration. The Equipment class is associated with the vaccines it stores, while the patients are related to the vaccines planned and already applied. The Digital Twin acts as a link between these elements, monitoring equipment, tracking patients, and managing vaccine administration.

This UML model organises the critical points related to the immunisation process in a primary health care unit in a systematic and integrated way, ensuring the proper conservation of vaccines through continuous monitoring and issuing timely alerts to prevent risks associated with temperature variation. It also improves the control of immunisers by recording information on expiry date, number of doses and application, reducing waste. The approach also boosts adherence to the vaccination schedule by providing automatic notification mechanisms for patients and health professionals, anticipating due dates, and reducing gaps in vaccination coverage. In this way, the model offers more precise support for strategic decision-making, based on data and predictive analyses, guaranteeing greater effectiveness and safety in vaccination campaigns and cold chain management.

## 4 Proposal for a DT architecture based on ISO 23247–the TWINVAX

In this work, the digital twin (DT) framework for manufacturing, standardised by ISO 23247, is used as a basis in a healthcare context. It is important to emphasise the suitability of the standard’s terms and domains for this specific use case. The proposal involves designing a DT implementation architecture aimed at temperature monitoring, which will include a 2D dashboard and a prediction space with anomaly detection and time series temperature prediction models. In addition, the architecture will have a module for counting doses applied by type and dose, allowing for cross-referencing with the estimate of vaccines needed for a given population. This will make it possible to conduct preventive vaccination actions. According to the ISO 23247 standard, the DT’s functionalities have different levels of maturity and complexity, including monitoring, remote access, simulation, command, control, optimisation, and predictive analysis, which enables effective feedback both from the end user’s point of view and from the equipment’s operation.

**Figure 5:**
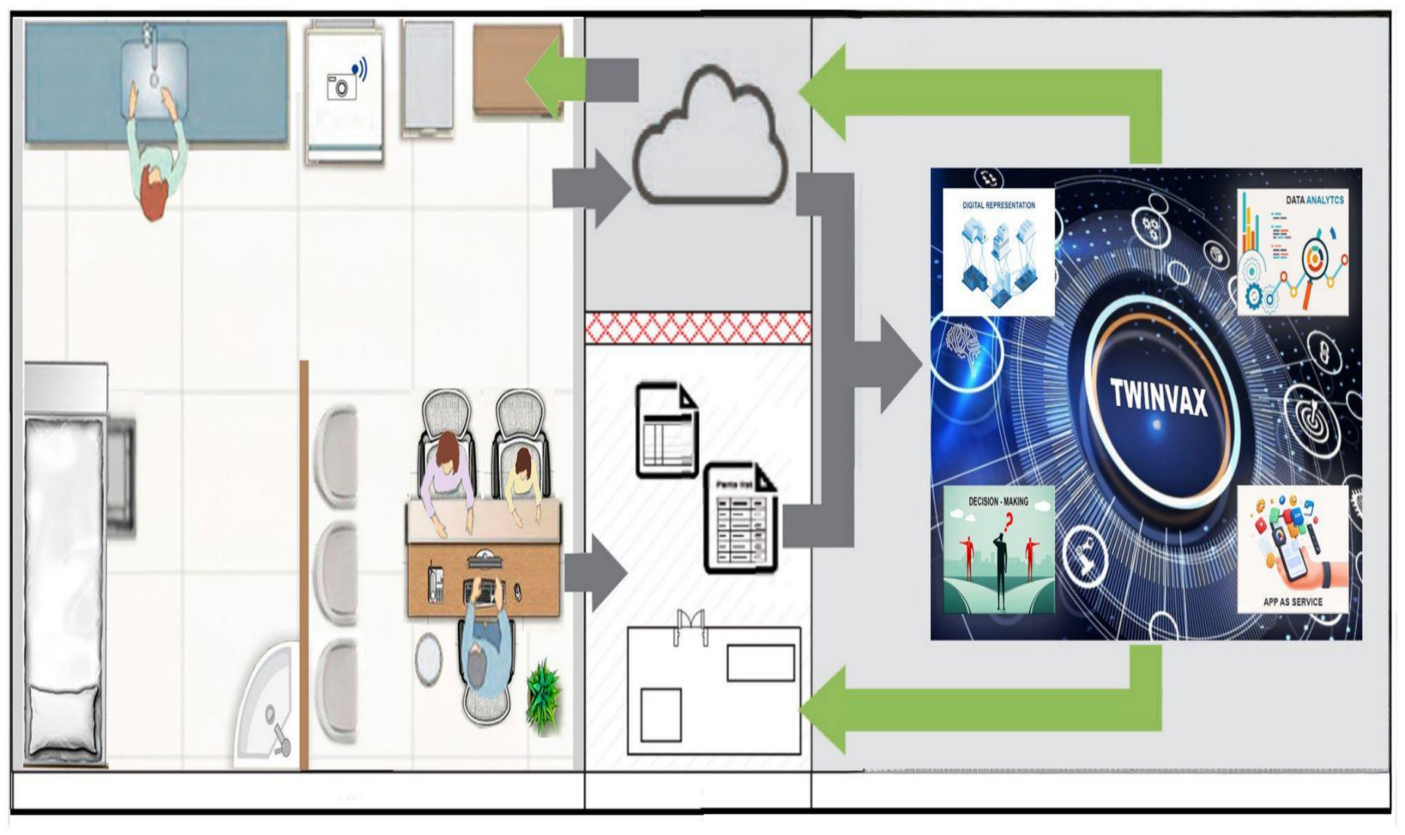
Illustration of the digital twin named TwinVax.

Observing the possibility of integration between IoT architectures and the digital twin modelling proposed in ISO 23247 and adapting them to a more simplified and understandable form for healthcare, a 4-layer architecture is proposed, capable of implementing immunisation DT, here, named TwinVax, as seen in Figure 5.

A Observable Manufacturing Elements - OME.

The first layer corresponds to the observable manufacturing element/entity domain, where the ILR and thermal boxes in use are located, from which temperature data will be collected. In addition, data will also be collected from the Electronic Health Records (EHR) on patient registration, vaccine records and application dates, which are essential for configuring the digital twin. This data will be recorded in electronic medical records and in databases of specific Immunisation Information Systems.

B Data Collection and Device Control Entity - DCDCE.

The second layer of the architecture corresponds to the domain of data collection and control of actuating devices present in the previous Observable Manufacturing Elements (OME) layer. This is the sensing layer, responsible for converting measurement quantities into digital data. Data extraction is conducted by a temperature sensor (example: DS18B20, DHT11 or LM35DZ) and the initial transformation takes place through local processing, using a device that converts the electrical signals into data transferable via a standard IoT integration protocol. As the sensing layer is based on IoT architecture, temperature sensors will be installed in ILRs and thermal boxes to detect variations outside the ideal range. These sensors will communicate with the edge layer via the 802.15.4 protocol. Temperature data will be collected hourly for 5 min, contextualised, and analysed by an algorithm to identify potential issues related to the loss of immunogenic power due to inadequate storage conditions. If the temperature exceeds 7°C or falls below 3°C, an immediate alert will be triggered. Regarding electronic records, the exchange of information will be made possible using the HL7 (Health Level 7) standard, which includes FHIR (Fast Healthcare Interoperability Resources) for more modern and flexible data exchange. FHIR is designed to facilitate the interoperability of health data (in this case, vaccination data), allowing easy integration of various healthcare systems and enabling more real-time data exchanges. Integration with HL7 and FHIR, involving the Electronic Health Record (EHR), the vaccination registration information system and the digital twin, enables efficient and secure communication between these systems. With these interoperability standards it is possible to synchronise clinical data and vaccination information, ensuring that patients’ medical records are always up-to-date and accessible. What’s more, with RESTful APIs (Representational State Transfer), developed according to HL7 and FHIR specifications, the digital twin will be able to continuously access and update this data, using machine learning algorithms to analyse and predict future vaccination needs. This makes it easier to implement proactive and personalised strategies for managing patients’ health. When it comes to protecting sensitive data, the use of security protocols such as OAuth 2.0 to authenticate and authorise access to APIs will enable only authorised users to access and manipulate this data, maintaining the integrity and confidentiality of the information. In addition to using the OAuth 2.0 protocol for authentication and access authorisation, it is essential to define the security approach about data encryption, both in transit and at rest, especially given the sensitive nature of the health information processed in the system. Encryption of data in transit will be guaranteed in this case using protocols such as TLS (Transport Layer Security), which ensures the integrity and confidentiality of information during transmission, protecting against interception and tampering attacks. On the other hand, encryption at rest implemented in databases and vaccination log files, using robust algorithms, will be AES (Advanced Encryption Standard), guaranteeing protection for sensitive stored data. In addition, the implementation of tokenization and data masking techniques can be considered to ensure that personal and confidential information is only accessed by duly authorised users, minimising the risks of data leakage.

Preferably, data will be collected once a day, at the end of opening hours. Considering the communication layer in the Digital Twin and IoT modelling, it is essential that the device chosen for signal conversion has good connectivity capacity. Therefore, the device selected for data acquisition, integrated with the temperature sensor in this case, is the ESP-32 (model WROOM-D), due to its ease of connection via Wi-Fi communication. A server application was developed using JavaScript technology in the Node.js environment, which is embedded on the ESP-32 board. The ESP-32 can be programmed using Node.js with support from the Espruino framework or via the NodeMCU, which allows JavaScript to be run on embedded systems. This flexibility provides an accessible development environment for creating automation and Internet of Things (IoT) applications. In order for the application developed with the ESP-32 to communicate with the refrigerator or cooler that has a temperature sensor, it can use protocols such as MQTT or HTTP for data transmission, taking advantage of the ESP-32’s Wi-Fi connectivity. The ESP-32 must be connected to the temperature sensor via GPIO pins and programmed to read the temperature at regular intervals. After reading, the data can be published to a server, allowing the application to receive real-time updates on the condition of the vaccine storage equipment. With this, the application can display the temperature, record histories, and implement notifications to warn of any variations that could jeopardise the integrity of the stored products.

The application communicates with the devices via Wi-Fi to collect relevant data, which is then made available to the Digital Twin domain through various TCP/IP sockets, utilising the MTConnect and MQTT application protocols, as recommended by ISO 23247. An MQTT broker receives the data from the devices, formats it, and makes it available to any interested subscribers. Simultaneously, a Node-RED service running on the ESP-32 Gateway acts as an MQTT subscriber, collecting the data from the broker and sending it directly to the cloud for storage and visualisation. Additionally, an MTConnect adapter is developed to receive data from the machine via TCP communication, transmitting this information to an MTConnect agent. This agent formats the data in XML and makes it accessible via a RESTful API for consumption by any interested web client, facilitating the integration and analysis of the information.

C Digital Twin Platform - DTP.

The third layer of the system is the Digital Twin Platform (DTP), which represents the core of the operations developed. This layer contains the application services and functionalities that serve both end users and other systems. To support this architecture, the computing infrastructure must provide various functions, including data analysis, aggregation, storage and the application of rules. Given the need for high availability and optimised processing, Amazon Web Services (AWS) was chosen as the platform for implementing the solution. This decision was based on the robustness and advanced functionalities that AWS offers as a cloud service, suitable for meeting the system’s requirements. The main functions performed include:

Communication: The cloud is responsible for receiving data from the previous layer and redistributing it among its internal services. To optimise network utilisation and reduce computational costs, especially on low-power devices such as the ESP-32, the MQTT protocol (55) was adopted. This protocol is widely recognised for its efficiency in IoT environments, allowing for light and effective communication.
Data Transformation: This function involves the conversion, translation and aggregation of data within the Processing layer of the IoT architecture and the Aggregation layer of the Digital Twin modelling. Through this transformation, essential metrics can be calculated, such as the average daily temperature and the percentage of vaccination coverage by vaccine type and audience. In order to streamline processing and facilitate data transformation, the AWS IoT SiteWise service, together with Node-Red, was selected, as indicated by Clerissi et al. (56). This approach ensures lightweight software focused on efficient data presentation.
Storage: To meet the demand for a data storage system with a low computational cost, databases such as InfluxDB or TimescaleDB have been suggested, which are designed to manage time series and are suitable for IoT applications (57). These databases enable efficient management of large volumes of data, which is essential for continuous monitoring of temperature and vaccination coverage.

The proposed architecture is defined for an isolated primary health centre, but when considering expansion to larger networks of health units or even national health systems, the system’s scalability becomes crucial. To ensure that the solution is scalable, an architecture based on microservices should be adopted, allowing different components of the system, such as temperature monitoring, vaccine management and patient records, to be updated, scaled and maintained independently. This approach makes it easier to expand the infrastructure in a modular way, without impacting the functioning of the existing parts. In addition, the use of cloud services such as AWS, already mentioned in the architecture, should allow for the dynamic expansion of resources according to demand. The elasticity of the cloud makes it possible to automatically allocate computing and storage capacity, allowing the system to adapt to the increase in data generated, such as the increase in sensors and vaccination units. In national health system scenarios, it is also important to consider the use of content distribution networks (CDNs) to guarantee the efficient and real-time delivery of information, as well as implementing load balancing mechanisms to distribute data traffic between servers, avoiding overload and guaranteeing high availability.

The services offered by TwinVax, as discussed in this paper, include temperature monitoring, accompanied by a 2D visualisation (dashboard) of the conditions of the ILR and thermal boxes, as well as predictive analyses based on Artificial Intelligence for monitoring vaccination coverage.

D User Domain - UD.

In this domain, we have those interested in consuming the services provided in the context of the Digital Twin, including the local health team and managers at local and regional levels. However, this approach can be extended to other management levels that require monitoring and simulation services. The system should be able to generate SMS (Short Message Service) messages to notify the health team and management of certain temperature conditions and the need for timely vaccination. The system must also send SMS messages to patients advising them of the correct dates for vaccination. In addition to the messages, visual alerts will be implemented for temperature and vaccination monitoring, with the creation of an interactive dashboard, like Grafana, using colours and icons to indicate the status in real time. In normal conditions, the colour green will be used, while yellow will be used for alerts and red for emergencies. In addition, it is possible to include pop-up notifications or banners that appear on the dashboard when an alert is triggered, ensuring that staff can quickly visualise the information. To increase robustness and speed of response, it is also advisable to consider notifications via mobile apps with push alerts, ensuring that information reaches staff instantly, regardless of their location. Finally, the installation of visual alarm systems, such as LEDs that change colour or flash in response to alerts, can provide immediate notification to those physically present on site, complementing the communication strategy.

As proposed, this layer fulfils two main functions:

(i) Data Visualisation: this is an important component of the Digital Twin model, allowing information to be analysed to generate insights, according to the Analysis, Insights and Action phases. We understand that Grafana’s good usability for data visualization in IoT systems makes this tool an appropriate choice for this function. However, it is crucial to note that Grafana must be properly configured to generate immediate visual alerts in cases of temperature variation, as well as providing a historical visualisation of the data, ensuring that teams can respond quickly to any anomalies detected.
(ii) Scenarios with Predictive Vaccination Analyses: This layer will present scenarios that offer predictive vaccination analyses. This will allow the healthcare team to anticipate the demand for vaccines and identify individuals who are close to their vaccination dates, making it easier to implement preventive strategies.

Recognizing the critical importance of data protection and regulatory compliance in digital healthcare systems, the TwinVax architecture adopts ISO/IEC 27001 as a foundational framework for information security governance. In alignment with international standards for information security management, the system incorporates practices designed to ensure the confidentiality, integrity, and availability of health-related data. Key mechanisms—such as role-based access control, encryption of data at rest and in transit, and multi-factor authentication—are consistent with the guidelines established in Annex A of ISO/IEC 27001, particularly controls A.9 (Access Control), A.10 (Cryptography), and A.12 (Operations Security).

Security governance within TwinVax is applied transversally across all architectural domains, with particular emphasis on the DTP and DCDCE domains. The adoption of ISO/IEC 27001 principles informs risk management procedures, access audit trails, and the safeguarding of sensitive information, ensuring that all implemented security measures reflect internationally recognized best practices.

By explicitly embedding ISO/IEC 27001 into the digital twin architecture, the TwinVax proposal not only strengthens the protection of patient data and supports compliance with national health data regulations, but also enhances traceability, accountability, and system reliability—key requirements for deployment in sensitive and high-stakes healthcare environments.

## 5 Results and discussion

Given the architecture developed, a temperature management solution was created for an immunisation service in a primary health care centre, focusing on more precise and rigorous control and monitoring of the thermal conditions for storing immunobiologicals. This ensures the maintenance of specific temperature ranges, guaranteeing the quality, safety, and immunogenic efficacy of vaccines. The proposed system allows for the collection, storage, monitoring and visualisation of temperature data from sensors installed in ILRs and thermal boxes. In addition, this same architecture includes surveillance of vaccination coverage by extracting data from information systems that record both the vaccines applied and the individuals vaccinated.

When exploring the details of this architecture, it is crucial to address fundamental issues for the data collection and management process within the context of a primary healthcare centre. The interaction between the physical service and its digital counterpart is not merely a passive replica, but an active interconnection, in which data and information flow in a bidirectional manner in order to optimise processes and decisions. Among the main questions that guided the design of TwinVax were:

- What would be collected from the service? The immunisation service can generate thousands of data. But what data would be needed for TwinVax? In this regard, it was decided that the data collected should include:

Temperature: Continuous temperature measurements of the ILR and thermal boxes, using appropriate sensors (such as DS18B20 or DHT11).

Patient data: Information from Electronic Health Records (EHR), covering registration, vaccination history, and administration dates of vaccine doses.

Vaccine information: Types, doses applied, and expiry records, which are essential for counting doses and forecasting vaccine needs. In addition, the current schedule of vaccines in the immunisation programme.

- When to collect? What would be the frequency of collection of thousands of data points, in real or near-real time, that the gateways and communication protocols support? What is the minimum mirroring capacity that the DT can have in relation to what the service can generate, updating and sending? For this purpose, it was decided that temperature data would be collected every hour for 5 min, while EHR data would be collected at regular intervals, in our case at the end of each service day. This time could be adjusted according to the needs of the healthcare team. However, in critical situations, such as abrupt temperature variations that could threaten the preservation of vaccines, temporarily increasing the frequency of data collection could be useful to obtain greater detail in real time. To do this, it would be important to monitor the impact of higher frequencies on network bandwidth and latency. Adjusting the frequency may become necessary in this case.
- How to collect? Will the reception flow be continuous, using buffers and batches, or will the data initially received have to be stored in a database? The data is collected via sensors connected to the ESP-32 device, which transfers the data via communication protocols such as MQTT or HTTP. This data is then formatted and sent to the cloud for processing and analysis. If the connection to the cloud is not available for an extended period, the ESP-32 must temporarily store the data in a local database (for example, using an inbuilt database library such as SQLite). In our case, the ESP-32 would collect the data from the sensors at the intervals already mentioned and store it in a local buffer. The buffer can be implemented as a queue (“First In First Out” -FIFO) to maintain the order of the data, allowing the device to collect and store the data even when the connection to the cloud is not available. Sending the data to the cloud could also be done in a hybrid format, also sending it in batches at regular intervals (for example, every 1 h or every 1,000 samples). In this case, the ESP-32 will have to check the buffer and send the accumulated data to the cloud.
- Where to store the data collected? Over time there will be a huge amount of data stored in the DT environment. Should it be stored in local files or databases, or in the Cloud? The data collected should be stored in the cloud, in a suitable database such as InfluxDB or TimescaleDB, allowing easy access and scalability. If there is no connectivity for long periods, the ESP-32 should store the data temporarily in a local database.
- How should the collected data be stored? Is there a reference data schema to be instantiated in the DT model? Should the data always be kept, or should it be ‘cleaned’ frequently? All of it or just some? The data should be organised according to the types of databases used, ensuring that the information is easy to access and consult, respecting data retention and cleansing policies.
- When and how to act in the immunisation service? According to the analyses carried out in the DT environment, physical action is required. In addition, depending on the seriousness of the situation, action must be immediate and carried out in real time. However, action on the physical system has numerous implications, it can incorrectly override the function of local supervision routines, and there are situations where this is not necessary or even unauthorised. What are the situations in which action can be taken on the job? What is the governance/compliance restrictions on permitting action? What and how can real-time restrictions be defined when immediate action is required? In the case of temperature monitoring and control, action must be taken immediately when alerts are generated by the system, such as temperature variations approaching the extremes of the ideal range. Healthcare staff should be notified via SMS or real-time notifications on the interactive dashboard. To ensure greater security, usage scenarios should be tested to verify the effectiveness of real-time notifications and assess whether staff can react appropriately. In the case of vaccination coverage surveillance, healthcare staff and managers should receive daily updates on which individuals need to be vaccinated in a timely manner, as well as which vaccines should be applied.

When discussing the DT user domain layer, we are essentially interested in visualising the operation of the immunisation service through its DT; accessing various types of DT information; and accessing service information. To do this, we need to consider.

- What and how to virtualise the immunisation service model? What to consider and how to model service mirroring? The service model should be virtualised using an interactive dashboard (such as Grafana) that represents the conditions of the ice lined refrigerator and vaccination coverage in real time. The model should include temperature data, vaccine stock, alert notifications, as well as information on individuals and vaccines to be received and received.
- What to store? Considering the different modules of the reference architecture and the possibilities of external access, what information should be stored for the demands of the various Clients? In this case, the storage would comprise historical temperature data, vaccination records and patient demographic information, allowing for analysis and report generation.
- What should be visualised? Which elements of the service should actually be visualised and their movements or changes of state shown? Users should see temperature graphs, alerts, and data on vaccination coverage at an individual and collective level, with a colour system to indicate the status of conditions.
- What to analyse and simulate? What to analyse during the operation/viewing of the service? What performance indicators and KPIs should be generated for management? What problems or events should be taken to the simulation level to further support predictive and prescriptive analyses? In the case of the proposed Digital Twin, analyses should include forecasting vaccine demand, detecting temperature anomalies and identifying patients who are close to their vaccination dates. Identifying individual vaccination needs should include: (i) Models to identify vaccine demand based on historical and demographic data, and (ii) Identification of vaccination coverage patterns to facilitate the timely anticipation of vaccination requirements.
- Where, when and how to act? What exactly should be allowed in terms of action? Who (people and systems) should be informed of each action and its effects? Action must take place on the basis of analyses and alerts generated by the system. The healthcare team should be notified immediately of any anomalies detected or the need for vaccination, using SMS and real-time notifications.

By addressing the critical issues listed and seeking concrete solutions, TwinVax presents itself as a fully viable approach, based on the continuous monitoring of essential conditions for vaccine conservation, combined with the optimisation of vaccine interventions in line with the current vaccination schedule. The system integrates advanced anomaly detection and time series prediction technologies, promoting data-based management to guarantee the safety and efficacy of stored immunobiologicals.

Strict temperature control in ILR and thermal boxes is one of the fundamental pillars of the system. Small temperature variations can jeopardise the immunogenic capacity of vaccines, generating significant losses. To mitigate this risk, TwinVax uses high-precision sensors integrated into the system, which ensure early detection of critical deviations, reducing losses and guaranteeing the integrity of immunobiologicals. Another significant distinguishing feature of TwinVax is its predictive module for analysing vaccination coverage. This functionality enables the health team to anticipate demands and optimise actions, minimising the need to actively seek out users with vaccine delays. Cross-referencing data in real time, such as the administration of doses and the schedule set out in the vaccination calendar, enables early immunisation interventions. This makes it possible to vaccinate the right people at the most opportune times, promoting greater adherence and vaccination coverage. The automatic alerts generated by the system for health professionals also play a crucial role, allowing individualised actions to be prioritised in the most vulnerable populations or areas. In this way, the impact of immunisation campaigns and the protection of public health are increased.

One of the key functionalities that could significantly enhance the TwinVax is the analysis of the risk of non-compliance with vaccination schedules, based on socio-economic and demographic variables. These variables—such as maternal education, family income, APGAR score at birth, birth complications, and place of residence—would be processed and weighted by machine learning (ML) algorithms. These algorithms would help predict the likelihood of an individual not adhering to the vaccination schedule, allowing for more targeted interventions.

Machine learning models such as decision trees, support vector machines (SVM), random forests, or gradient boosting could be employed to develop a predictive model. The system would continuously refine its predictions by incorporating real-time health data, vaccination history, and socio-economic factors. This dynamic adaptability would enable the digital twin to offer timely and accurate risk assessments.

Cloud-based AI platforms would facilitate secure data processing, ensuring compliance with privacy regulations like GDPR, while supporting the scalability of the model. The predictive model would not only help in monitoring vaccination status but also in designing interventions such as personalized reminders or outreach by health educators to improve vaccination uptake.

These machine learning algorithms would rely on key predictors like antenatal care visits, institutional deliveries, maternal health, and socio-economic status. By focusing on these factors, the model could predict which individuals are at a higher risk of not following their vaccination schedule, facilitating interventions that are tailored to specific needs. This approach would allow healthcare providers to target resources more effectively, improving vaccination coverage rates and reducing the risk of preventable diseases.

Although it wasn’t the focus of this first phase of development, TwinVax also has potential for optimising vaccine stocks. By correlating data on predicted demand with existing stocks, the system could reduce waste and prevent shortages of immunobiologicals. This future perspective reinforces TwinVax’s role as a comprehensive and strategic solution for immunisation management.

Another point to be addressed with the creation of TwinVax would be its concrete contribution to improving the patient experience. Integration with electronic health records enables proactive communication, sending SMS alerts to remind patients of their vaccination dates. This direct engagement with the population could lead to greater adherence to the vaccination schedule, especially in areas with higher rates of forgetfulness or resistance. Regarding professionals, the proposed platform would provide an efficient visualisation interface for health managers, allowing them to monitor data in real time using interactive dashboards such as Grafana. This functionality would promote evidence-based decision-making, both in temperature monitoring and in the effectiveness of vaccination actions. The clear and immediate visualisation of alerts and critical indicators would also facilitate the adoption of rapid corrective measures, reducing possible delays in responding to risk situations. In this way, the platform presents itself as a valuable tool for health professionals by providing accurate and timely information, which is fundamental for the effective management of services and the effective tackling of epidemiological challenges.

The adoption of the TwinVax digital twin would enable significant advances in the coordination and governance of immunisation efforts. Its expanded capacity to generate predictive scenarios would not only improve resource management but also provide a solid foundation for implementing more targeted and effective public health policies. Analyzing historical data would allow vaccination strategies to be tailored to local needs, ensuring more effective coverage and better health outcomes for the population. In this context, TwinVax presents itself as a robust and innovative solution for modernizing Primary Health Care processes. Expanding this discussion will certainly require evaluating the strategic and operational benefits that the platform can offer to both healthcare professionals and the populations they serve.

Despite the strategic and operational advantages that TwinVax can offer, there are some limitations to its implementation. One of the key challenges is the complexity of integrating diverse data systems, such as Electronic Health Records (EHR) and IoT-connected temperature sensors. While protocols like HL7 and MQTT facilitate this integration, the heterogeneity of devices and standards across different healthcare units presents technical obstacles. Moreover, real-time data collection and transformation can be affected by connectivity issues and bandwidth limitations, particularly in remote areas, leading to delays in synchronizing information and, consequently, in generating alerts. The absence or delayed registration of vaccination records also represents a significant limiting factor.

The challenges encountered for implementation also include adapting an architecture originally developed for manufacturing (ISO 23247) to the public health context. Adaptation involves the need to simplify certain concepts, such as manufacturing-specific terminology, into language that is more accessible and applicable to immunisation and vaccine monitoring. In addition, the need to guarantee the security and privacy of data, especially patients’ health records, is certainly the biggest challenge, since any healthcare system must adhere to strict privacy regulations, such as the GDPR in Europe.

Training healthcare staff to fully utilise the tools offered by TwinVax is also challenging. Implementing new technologies requires not only technical integration, but also a learning curve for the professionals who use the system on a daily basis. The dashboard’s user-friendly interface and automated notifications would help minimise this challenge but adapting to the new workflow can still be slow in regions where familiarity with digital technologies is limited.

TwinVax’s sustainability and scalability are also critical points. Although the system has shown great potential, its expansion to a more complex context, with different types of vaccines and varying logistical conditions, requires financial and technological resources. The cost of implementing the IoT infrastructure and cloud storage, as well as ongoing maintenance, are factors that need to be carefully considered by health authorities when deciding to expand the project. In short, despite the challenges and limitations, TwinVax is proving to be a valuable tool for boosting immunisation, especially in terms of proactive monitoring and expanding vaccination coverage.

Governance and compliance will be key elements in the implementation of TwinVax, especially due to the sensitivity of health data involved. To ensure that the system complies with privacy regulations, such as the General Data Protection Regulation (GDPR), all patient data interactions will be protected with encryption and strict access control measures.

Alongside GDPR and the AI Act, the TwinVax framework is designed to conform with international standards for information security management, notably ISO/IEC 27001 and ISO/IEC 27799, which outline best practices for protecting personal health information. These standards guide the implementation of robust controls for confidentiality, integrity, and availability of data throughout the system—from sensor-level acquisition to cloud-based analytics. By incorporating these principles, TwinVax reinforces resilience and trust in its data protection mechanisms.

The proposal is also aligned with recent regulatory developments in the European Union, particularly the Artificial Intelligence Act (AI Act), which categorises healthcare-related AI systems as high-risk and introduces a comprehensive framework for their ethical and lawful deployment. The AI Act emphasizes transparency, explainability, and human oversight—principles embedded in the design of TwinVax. These are in harmony with the European Commission’s ethical guidelines for trustworthy AI, which advocate for human agency, technical robustness, privacy and governance, transparency, non-discrimination, social well-being, and accountability.

To support these principles, TwinVax includes auditing and traceability mechanisms that allow alerts and decisions—such as those triggered by cold chain breaches or missed vaccinations—to be reviewed, validated, and explained. This approach not only meets compliance standards but also helps cultivate public trust, particularly in settings where automated systems influence clinical or public health decisions.

Securing the data managed by TwinVax is paramount. Access will be limited to authorized users, and exchanges with external systems, such as Electronic Health Records (EHRs), will occur through secure, auditable APIs. This architecture guarantees data is accessed appropriately and fulfills the transparency and security requirements expected by regulatory frameworks.

Beyond data privacy, the accurate interpretation of insights generated by the digital twin plays a key role in enhancing immunisation strategies. Real-time monitoring of immunobiological storage conditions ensures vaccine integrity, reducing the risk of diminished efficacy. More importantly, TwinVax empowers health professionals with data to make timely and informed decisions, such as predicting the likelihood of missed appointments due to behavioural or social factors. Anticipating such risks enables proactive, targeted interventions that can boost vaccination coverage and protect vulnerable populations more effectively.

Finally, the ethical issues surrounding the use of a digital twin for the immunisation service cannot be ignored. The system must be designed to serve all population groups equitably, avoiding any discrimination in access to vaccines. In addition, automated decision-making, such as targeting vaccination campaigns based on coverage forecasts, must be done with human supervision to ensure that social and cultural aspects are considered. Transparency in the operation of the digital twin and clear communication about how patient data is used are essential to building trust in the adoption of this technology, ensuring that it makes an effective contribution to public health.

## 6 Conclusion

The use of digital twins mainly in process optimization and healthcare lines presents important challenges related to data integration, privacy, and interoperability. However, trends indicate great potential in personalising treatment, prevention, remote monitoring, informed decision-making, and process management, which can result in significant improvements in quality and efficiency in healthcare. This work could, in some way, contribute to expanding discussions on the topic, opening space for new reflections.

It is worth noting that, despite the sector’s rising costs, increased demand for services and the already established role of primary health care in improving the health levels of a population, there was a lack of studies in the present literature, on the specific use of digital twins. As it is the main axis of changes in health systems to improve health levels, primary health care (PHC) can receive due attention from the scientific community and the industrial complex as a promising field for the use of twin digital technology in healthcare and in the operation of its units.

The deployment of a Digital Twin for an immunisation service represents a breakthrough in public health management, particularly with regard to ensuring the immunogenic potency of vaccines. This model efficiently integrates three fundamental components: the equipment used for vaccine conservation, the vaccines themselves, and patient data. The Digital Twin’s ability to issue real-time alerts about temperature variations is a significant advancement in the preservation of immunobiologicals. The proactive management of storage conditions, along with rigorous monitoring, can significantly reduce the risk of vaccine deterioration, thereby enhancing the effectiveness of immunisation programs. Additionally, the ability to conduct scenario analyses to identify groups at risk of missing vaccination underscores a data-driven approach that can transform vaccination strategies, enabling a more targeted and efficient response to population needs.

The digital twin enables the definition of minimum standards necessary to ensure that vaccines are stored and managed appropriately, ensuring that immunisations are carried out safely and efficiently, thereby minimising the risk of compromising the effectiveness of vaccines administered to patients and, consequently, reducing risk to patients and the waste of costly medicines. Furthermore, by estimating the required quantity of vaccines based on vaccination schedules, it could not only streamline immunisation logistics, but also reduce waste, ensuring that health services are well equipped to effectively respond to demand.

The development of the present work enables the application of some Industry 4.0 technologies, such as the Internet of Things, Cloud Computing, Artificial Intelligence, and Digital Twins. The developed architecture provides a significant contribution in terms of the generality of the implemented communication protocol and the use of open-source tools, utilising freely available knowledge from different sources, existing and popular network technologies such as Wi-Fi, and low-cost devices like the ESP-32 WROOM-D microcontroller. This allowed for the modelling and development of a Digital Twin based on the ISO 23247 architecture, aimed at the extraction, monitoring, storage, and visualisation of historical and instantaneous temperature data from vaccine storage equipment in an immunisation room, as well as patient and vaccine data through integration with a prediction module, bringing Industry 4.0 technologies into the healthcare field and exploring their application in other domains.

This work demonstrates the flexibility of the architecture, particularly the possibility of integrating different types of applications based on the available communication protocols. Other modelling tools, 3D visualisation, data analysis, and even other types of I/O hardware can be used within the developed architecture. Additional modules can be added as needed, and it is also possible to use only a subset of the constituent modules when convenient, without compromising the overall system.

As a suggestion for improvements in future research, the use of the prediction module for data analysis and insights in Digital Twin modelling, specifically in the enhancement of vaccine inventory control in the immunisation room, is proposed. The system would contribute to efficient vaccine inventory management, reducing waste.

Although the current stage of the project focuses on conceptual development and simulation using synthetic data, the proposed architecture is being prepared for real-world validation. In the next phase of the research, this simulation will be complemented with real data from a pilot implementation currently underway in the state of Rio de Janeiro, Brazil. This case study will allow for an in-depth evaluation of the system’s performance in an operational immunisation setting and provide concrete evidence of its applicability and benefits within public health environments.

Building on this foundation, future studies should explore the consolidation of Digital Twin applications in healthcare—particularly in processes related to primary healthcare—by identifying and strengthening key initiatives necessary to sustain and scale the progress achieved. These efforts will be essential to guide the adoption of such technologies, support decision-making, and ultimately contribute to the resilience and responsiveness of health systems.

In conclusion, the proposed TwinVax Digital Twin architecture enables the effective collection, monitoring, and analysis of vaccination and temperature data. This not only ensures the safety of the vaccines but also enables preventive actions, contributing to the qualification of immunisation efforts in a given territory. The integration of IoT technologies and predictive analytics provides a robust and innovative solution for the healthcare sector, particularly in Primary Healthcare. In a global context of challenges, such as epidemics and disease outbreaks, the use of Digital Twins strengthens the response capacity of health systems, promoting a promising future for healthcare, especially in immunisation.

## Data Availability

The original contributions presented in the study are included in the article/supplementary material, further inquiries can be directed to the corresponding authors.
